# Developing a Social Autopsy Tool for Dengue Mortality: A Pilot Study

**DOI:** 10.1371/journal.pone.0117455

**Published:** 2015-02-06

**Authors:** María José Arauz, Valéry Ridde, Libia Milena Hernández, Yaneth Charris, Mabel Carabali, Luis Ángel Villar

**Affiliations:** 1 School of Public Health (ESPUM), University of Montreal / University of Montreal Hospital Research Center (CRCHUM), Montreal, Canada; 2 Centro de Atención y Diagnóstico de Enfermedades Infecciosas (CDI-APRESIA), Bucaramanga, Colombia; 3 Dengue Vaccines Initiative / International Vaccines Institute, Seoul, Republic of Korea; 4 Universidad Industrial de Santander, Bucaramanga, Colombia; Rajarata Univeresity of Sri Lanka, SRI LANKA

## Abstract

**Background:**

Dengue fever is a public health problem in the tropical and sub-tropical world. Dengue cases have grown dramatically in recent years as well as dengue mortality. Colombia has experienced periodic dengue outbreaks with numerous dengue related-deaths, where the Santander department has been particularly affected. Although social determinants of health (SDH) shape health outcomes, including mortality, it is not yet understood how these affect dengue mortality. The aim of this pilot study was to develop and pre-test a social autopsy (SA) tool for dengue mortality.

**Methods and Findings:**

The tool was developed and pre-tested in three steps. First, dengue fatal cases and ‘*near misses*’ (those who recovered from dengue complications) definitions were elaborated. Second, a conceptual framework on determinants of dengue mortality was developed to guide the construction of the tool. Lastly, the tool was designed and pre-tested among three relatives of fatal cases and six near misses in 2013 in the metropolitan zone of Bucaramanga. The tool turned out to be practical in the context of dengue mortality in Colombia after some modifications. The tool aims to study the social, individual, and health systems determinants of dengue mortality. The tool is focused on studying the socioeconomic position and the intermediary SDH rather than the socioeconomic and political context.

**Conclusions:**

The SA tool is based on the scientific literature, a validated conceptual framework, researchers’ and health professionals’ expertise, and a pilot study. It is the first time that a SA tool has been created for the dengue mortality context. Our work furthers the study on SDH and how these are applied to neglected tropical diseases, like dengue. This tool could be integrated in surveillance systems to provide complementary information on the modifiable and avoidable death-related factors and therefore, be able to formulate interventions for dengue mortality reduction.

## Introduction

“They let my little girl die because I didn’t have 30 million [Colombian] pesos [≈15 000 USD],” says the grandmother of a 5-year-old child who died from dengue fever, a 99% avoidable cause of death [1,2]. Dengue fever is a viral disease transmitted between humans by *Aedes* mosquitoes [3]. It is a dangerous and debilitating disease where its manifestations can range from an influenza-like disease to a severe, life-threatening illness [1]. Tertiary level care is required for severe dengue management [1,2], which is often beyond the reach of many people at risk [1]. Though the global aim for dengue mortality is to reduce it by at least 50% by 2020 [4], until now, there is neither a specific treatment for dengue nor a licenced vaccine available [3]. Furthermore, vector control strategies-where current efforts lie- do not always provide satisfactory results [3]. As a result, dengue represents the most important arthropod-borne viral disease of public health significance [1].

In recent years, the annual average number of severe dengue cases (formerly known as dengue haemorrhagic fever) has grown dramatically [5], which in turn, increases dengue mortality [1]. In the Americas alone, 1.6 million dengue infections were reported in 2010, where 49 000 cases were severe dengue [6]. Severe dengue is a leading cause of serious illness and death among some Latin American countries [6].

Dengue epidemics pose a significant burden [2], similar to the burden of other childhood and tropical diseases, including tuberculosis [5,7]. In 2009, an average episode represented 18.9 lost days for hospitalized patients and 14.8 lost days for ambulatory patients [8]. The estimated global burden of dengue is approximately 0.6 million disability-adjusted life years (DALYs), with an estimation of 69 000 DALYs in the Latin American and Caribbean region [9].

In Colombia, dengue fever is a public health priority due to its re-emergence and its intense increasing transmission [10,11]. Dengue outbreaks have an exponential trend, in other words, each epidemic exceeds in magnitude the last epidemic [10]. After the largest epidemic of 2010, Colombia has recently experienced an outbreak of dengue in 2013 with 126 425 cases, representing an increase of 136% in relation to 2012 [12] with certain departments being particularly affected. This is the case for the department of Santander where the cumulative incidence, 1024.8 dengue cases per 100 000 inhabitants, is significantly higher than Colombia’s (478.3 per 100 000) [12]. Because dengue is essentially an urban disease, the population of Bucaramanga (Santander’s capital) is particularly at risk. Santander’s case fatality-rate is more than twice (5.4%) [12] the expected case fatality-rate for severe dengue (<2%) according to Colombia’s national millennium development goals [10], meaning that there is an excessive number of dengue-related deaths.

Dengue is a complex disease and its transmission depends on multiple factors involved at different levels. Global population growth and unplanned urbanisation result in substandard housing and inadequate water supply and waste management services, which promote dengue transmission [1,13]. Furthermore, different serotypes, strains, and genotypes of virus move between regions due to migration and globalization [13]. However, to date, the determinants of dengue mortality are not completely understood, especially the social ones. Very few studies have been interested on social determinants of dengue prevalence [14–16] and dengue mortality [17–19]. Two of them [17,18] study the social determinants of dengue mortality and measure quantitative variables from an epidemiological point of view, missing qualitative variables like the narration of the disease or perceptions of quality of care. On the other hand, Figueiró and colleagues’ study [19] evaluates quality of care and access to health services associated with dengue-related death using qualitative and quantitative methods. Still, little is understood concerning the path to death and how social determinants of dengue mortality are involved in health care use.

### The Social Determinants of Health (SDH)

The World Health Organization (WHO) Commission on Social Determinants of Health (CSDH) describes health inequalities as the “unfair and avoidable differences in health status seen within and between countries” [20]. Differences in mortality and morbidity rates depend on people’s socioeconomic position (social class, gender, ethnicity, education, occupation, income) for almost every disease [21]. These socioeconomic positions have an effect on health outcomes via people’s material circumstances, behavioral and biological factors, and psychosocial factors; altogether the SDH [21]. The SDH, comprising the health system, shape health outcomes (including mortality) [21]. There is a growing interest on the study of SDH and dengue fever [22–24], nevertheless it is not yet understood how the SDH affect dengue mortality.

Understanding the social determinants of dengue mortality is a necessary step in order to define interventions before taking actions to prevent the related avoidable deaths. Developing a tool that is able to identify the social determinants of dengue mortality could be a useful method, asked and needed by fieldworkers, to provide information that we wouldn’t be able to find elsewhere.

### Social autopsy as a death review method

Death reviews are useful to examine the immediate and contributing causes leading to death, and inform the health sector to ultimately prevent future morbidity and mortality [25]. Death reviews have been extensively used in the study of maternal mortality [25]. Methods used include: hospital-based maternal death reviews (see the definition in “Beyond the Numbers” [25] report developed by WHO); and, verbal autopsies, which determine the causes of death and other related factors that may have contributed to the deaths in women who died outside a health care facility [25]. Social autopsies have been used as a complementary interview method to verbal autopsies in order to explore the social, behavioral and health systems determinants of maternal and child deaths [26,27], in order to establish a “social diagnosis” of the contributing causes of death [27]. Social autopsy data is useful to identify modifiable factors present in the home, community, and health system in order to inform policies and practices [26]. Inspired by this method, we developed, what to our knowledge is the first social autopsy tool to study the social determinants of dengue mortality.

Because this is the first social autopsy tool developed and used for dengue mortality, it was necessary to conduct a pilot study to assess its practicality before applying it in the main study. (The main study aims to examine the social determinants of dengue mortality in Colombia). It was indispensable to pre-test the tool in the study context and make the necessary changes in case it was to be found “inappropriate or too complicated” [28]. One of the functions of pilot studies is to develop and test the adequacy of research instruments [28]. Publishing the results of pilot studies provides information on tools’ utility and may help saving resources from being spent on studies that may not yield reliable and valid results [29]. As a matter of fact, van Teijlingen and Hundley [28] discuss that pilot studies tend to be “under discussed, underused and under-reported”, therefore it is encouraged for researchers to report their pilot studies and the improvements made to the study design. To conclude, the aim of this pilot study was to develop and pre-test a social autopsy tool for dengue mortality in Colombia.)

## Methods

### Study population

The pilot study population consisted on dengue fatal cases (confirmed by virological, serological and/or pathological tests) and near miss dengue cases (those who survived from life-threatening dengue complications) hospitalized from January 2011 to November 2013 in the Santander department, in Colombia, regardless of age or sex. An inclusion criterion for both cases was hospitalisation, because dengue cases with complications or life-threating conditions should be hospitalised [2].

The social autopsy tool for dengue mortality was developed and pre-tested in three stages:

1. Development of the definition of a fatal case and a near miss in the context of dengue

The tool was intended to be applied to (the relatives of) fatal dengue cases and near misses, therefore it was necessary to define this population. Clear criteria must be established to be able to confirm dengue as the cause of death; hence, the importance of defining a fatal dengue case. On the other hand, a near miss is a concept that originated in the maternal health context. A near-miss case is defined as “a pregnant woman with severe life-threatening conditions who nearly die[d] but, with good luck or good care, survive[d]” [30]. Therefore, it was necessary to adapt this concept to the dengue context.

In the case of maternal health, where few maternal deaths took place, it is recommended to accumulate fatal cases over a period of years in order to have a better portrait. It is also helpful to use alternative outcomes, such as near-misses [25]. Using near misses in a death review context has several advantages. First, a near-misses review helps identify the factors that contributed to recovery, which can be comparable to studying the factors that contributed to death [30]. Second, in the case of maternal mortality, because obstetric complications occur much more frequently than maternal deaths, taking into account a larger number of cases allows a more detailed review of determinants [25]. This is also the case for dengue, where deaths remain relatively small in Santander [12], but of public health importance.

In order to define a fatal case and a near miss in the context of dengue mortality, we reviewed the literature (WHO guidelines, grey and published literature and local epidemiological reports) on the concepts of fatal case in dengue (characteristics of death, disease classifications, and definitions of fatal cases) and of near misses in maternal health. The team’s clinicians and public health researchers validated the definitions by corroborating them with their clinical knowledge and experiences.

2. Development of a conceptual framework on determinants of dengue mortality

Since a conceptual framework on social determinants of dengue mortality has not yet been published, it was necessary to develop one. The objective of building a conceptual framework was to rely on a pragmatic model, adapted to the Colombian context and able to guide the construction of a social autopsy tool; taking into account the complexity of the factors involved in dengue mortality (social, epidemiological, individual variables).

We conducted a literature review on conceptual frameworks used in dengue mortality and in other relevant health domains (maternal and child deaths), as well as on social determinants of health conceptual frameworks. The conceptual framework was built in three steps: 1) We based our model on Gabrysch and Campbell’s 2009 model [31] on delay phases and factors affecting use of delivery care and maternal mortality (adapted from Thaddeus & Maine’s “three delays” model); 2) We added some new variables of interest from Gabrysch and colleagues’ 2011 model [32], based itself on the 2009 model; 3) We added variables from the scientific literature associated to severe dengue and mortality. Finally, the model was adapted to the Colombian health context; particularly using the “four delays” Colombian model [33] to adapt the types of delays. The research and professional team involved in the project supported the development process by validating the added variables with their expertise.

3. Development and pre-test of a social autopsy tool for dengue mortality

**Development of the tool.** The construction of the social autopsy tool was based on the conceptual framework that was built and on other social and verbal autopsy tools (for maternal and child deaths) [27,34–37]. Variables from this model were operationalized in order to understand how they could be studied by the tool we propose. To this end, a table (S1 Appendix) was created with the dimensions and its indicators (operationalized variables), and the appropriate method to study each indicator (interview, observation, medical records or other). All components of the table were discussed among the research team until all agreed that the match between indicators and methods was consistent with the model and the targeted social determinants.

For practical reasons, the tool was designed as a multi-section questionnaire with the intention to serve as a structured interview guide. The tool was adapted according to the case: fatal case (interview with next of kin) or near miss (interview with survivor), adult or minor (interview with carer).

**Pre-testing the tool.** The questionnaire was pre-tested among next-of-kin of three fatal cases, and six near misses in the metropolitan area of Bucaramanga (Colombia) between November and December 2013. Access to fatal cases records was obtained with the authorization of Santander’s Department of Health. Near- misses records were obtained through the Industrial University of Santander’s Hospital. Fatal cases and near misses were established as dengue confirmed cases, based on lab tests, by the Santander’s Department of Health, and by the University Hospital, respectively. Participants were contacted by telephone in order to explain the objective of the study and to ask for their participation. Not all of the participants answered as some of them had changed telephone numbers or moved out. However, all of the participants that answered our telephone call accepted a home-visit to participate in the study.

**Ethical considerations.** The pilot study protocol was approved by the *Centro de Atención y Diagnóstico de Enfermedades Infecciosas (CDI)* Ethics Committee (Bucaramanga, Colombia). Written informed consent was obtained from all participants before the interview. In the case of minors, an informed written consent was obtained from the next of kin.

**Practicality criteria.** The pilot study had the objective of developing a social autopsy tool and making sure it could be easily administered in a Colombian dengue mortality context, which is the main focus of pilot studies [38]. For this, we assessed several aspects of the questionnaire: i) adequacy of the time needed to administer the questionnaire (explaining the study objective, obtaining consent, sections’ length); ii) the type of questions (open-ended, semi-open, and closed-ended questions); iii) the range of pre-formatted responses; iv) the sequence of questions (for fluidity); v) the difficulty in asking and understanding a question (uneasiness, taboos, etc.); vi) avoidable questions; and vii) the means to establish a trust relationship (relatively) with the interviewee (particularly for a fatal case). The tool was administered to pilot subjects in the same way it will be done in the main study. The necessary modifications were made after revision with the research team, and then it was piloted again until there were no modifications left. These procedures are known to improve the internal validity of a questionnaire [28].

**Face validity and content validity.** For face validity, the two persons administrating the tool gave their opinion on the validity of the tool and whether it measured what it was supposed to measure. They proposed amendments to the tool when necessary. In terms of content validity, as it was mentioned before, the tool is based on a robust model (conceptual framework) and on other social and verbal autopsy questionnaires. The conceptual framework, the table with the operationalized variables and the tool itself were reviewed by a team of experts on dengue fever and on social determinants of health. Required changes were made to the tool until a consensus was reached on the proposed questions and the operationalized variables from the conceptual framework.

## Results

### 1. Definition of a fatal case and a near miss in the dengue context

The fatal case and near miss definitions we propose are based on WHO’s Dengue Guidelines for Diagnosis, Treatment, Prevention, and Control [2], on the pregnancy continuum between extremes of normal and death [39] and on the research team’s expertise. These definitions are based on clinical criteria (i.e. dengue diagnosis) and on case management criteria (i.e. type of treatment).

**Fig 1:**
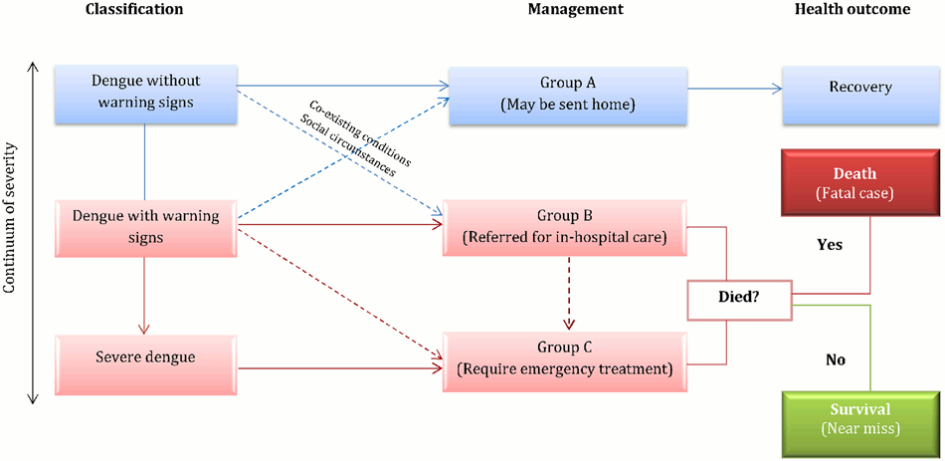
Fatal case and near miss definitions based on severity of illness, diagnosis, management treatment criteria and health outcome.

The general definitions obtained were as follows:

- Fatal case: the death of a hospitalised dengue case where diagnosis has been confirmed pre or post mortem (by autopsy, histopathology, viral tests and/or serologic tests). See table 1 for the specific definitions.
- Near miss: a dengue case with warning signs who has been referred for in-hospital care (Group B) or who has evolved into a severe dengue and requires emergency treatment (Group C) [2] (see Fig. 1). See table 2 for the specific criteria definition.

**Table 1:** Fatal dengue case definition.

Rarely seen	Scenario #1 (Gold standard)	Death of a hospitalised dengue case with a pre and/or post mortem confirmed diagnosis by autopsy, histopathology, viral tests and serologic tests.
Not often seen	Scenario #2	Death of a hospitalised dengue case with a pre or post mortem confirmed diagnosis by serologic tests; with an incomplete autopsy, but with a positive viscerotomy (spleen or liver biopsy); histopathology, and PCR.
Often seen	Scenario #3	Death of a hospitalised dengue probable case confirmed post mortem by a micro and macro compatible pathology, but with no concluding viral tests.
Usually seen	Scenario #4	Death of a hospitalised dengue case with no autopsy, but with pre or post mortem positive viral tests (PCR or viral isolation) or positive serologic tests (IgM).

**Table 2:** Criteria for a dengue near-miss case definition.

Probable dengue case with one or more warning signs
Abdominal pain or tenderness
Persistent vomiting
Clinical fluid accumulation
Mucosal bleed
Lethargy, restlessness
Liver enlargment >2 cm
Laboratory: increase in HCT concurrent with rapid decrease in platelet count
Or severe dengue case with one or more of the following criteria
Severe plasma leakage leading to:
Shock (DSS)
Fluid accumulation with respiratory distress
Severe bleeding as evaluated by clinician
Severe organ involvement:
Liver: AST or ALT > = 1000
CNS: Impaired consciousness
Heart and other organs
And one or more of the case management criteria
Hospitalization
Referred for in-hospital care (Group B) or requires emergency treatment (Group C)

With respect to Fig. 1, warning signs are what differentiates a dengue case with complications versus a non-complicated case that may be sent home (group A). In fact, warning signs require strict observation and medical intervention. See table 2 for a list of warning signs.

As Fig. 1 depicts, fatal cases and near misses resemble at the moment of arrival to the hospital: they are hospitalized dengue cases with warning signs or severe dengue cases. However, they differ on the health outcome (death or survival).

### 2. Conceptual framework on determinants of dengue mortality

The conceptual framework was developed with the purpose of understanding the underlying circumstances of health care use from the onset of dengue symptoms till mortality. This is relevant to our context because the common inclusion criterion for a fatal case and a near miss is hospitalisation. The model is inspired on a “three delay” expanded model [31], in order to take into account the delays associated with health care use. We also integrated social dimensions that influence behaviors and health outcomes from other conceptual frameworks on social determinants of health [21,40].

**Fig 2:**
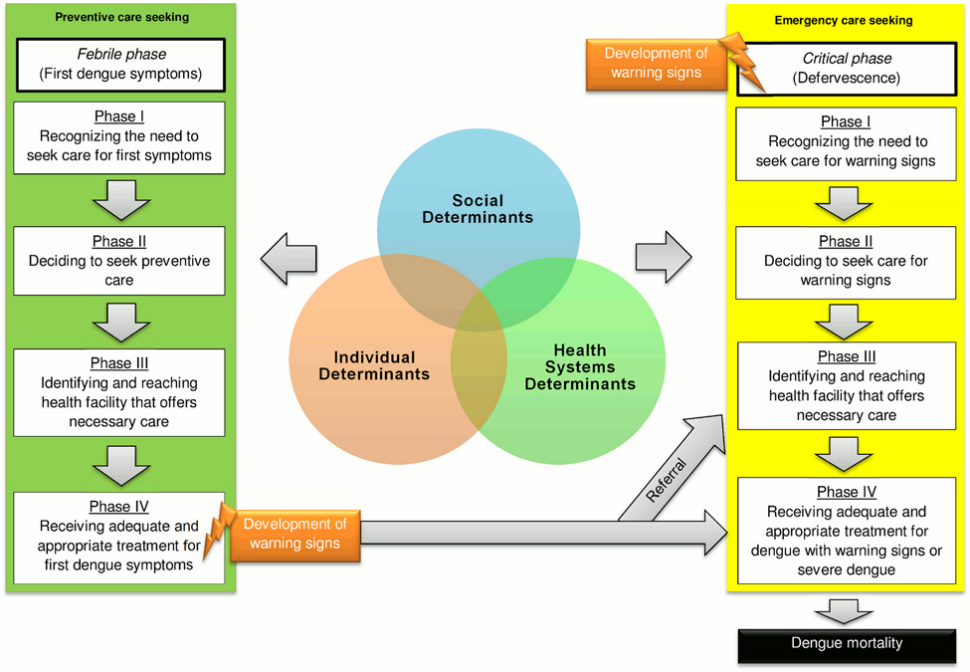
Conceptual framework on determinants of dengue mortality, based on Gabrysch and Campbell’s (2009) three delays expanded framework, on the literature on the determinants of dengue mortality, on conceptual frameworks on the social determinants of health and on the Colombian four delays model.

In the first place, the conceptual framework takes into account preventive care-seeking (for the manifestation of the first dengue symptoms) as well as emergency care-seeking (for dengue-related complications). One of the limitations of the “three delay” model is considering emergency care-seeking only [41]. Hence, the proposed model considers factors related to dengue mortality, even before the onset of warning signs (dengue-related complications). These two mechanisms however, are not independent. A dengue case may present warning signs while receiving preventive care. Then, the person will have to be transferred to another facility in order to obtain emergency treatment, which can involve once again, a third delay concerning the arrival to the referred facility (see Fig. 2).

Secondly, the delays for health care use were adapted to the Colombian “four delays” model [33]. The “four delays” model is an adaptation of the “three delays” model which divides the first delay in two: recognizing the need for care, and taking the decision to seek for care [33]. Moreover, the third delay is more elaborated. It refers to the delay of going to a health facility that provides *necessary care* [33]. Providing necessary care in the Colombian context implies two conditions. First, the level of care of the health facility needs to correspond to required care. For example, tertiary level care is needed for severe dengue [1,2]. Second, Colombians with a health insurance can only attend certain health facilities that are authorized to provide care by previous agreement with health insurance companies [42].

Finally, the conceptual framework describes three categories of the determinants affecting the delays for health care use related to dengue mortality: the social, the individual and the health systems. In our case, we used an “Individual” category instead of the “Behavioural” category described in the social autopsy [26], in order to include other non-controllable individual factors such as the biological ones. Overall, the variables within these categories are the factors that we consider are related to dengue mortality.

**Revision of factors related to dengue mortality.** Several factors have been identified as being related to dengue severity, which is an indication for factors related to dengue mortality. Among individual factors there is age [1,3,17,43–45], sex [1,3,5,13,17,44,45], “ethnic group” or “race” [1,17,43], education level [14,17], socioeconomic status [1,13,14,16], urban or rural place of residence [17], pre-existing chronic diseases [13,43], and secondary infection [3,13,16,18,43–45] (which is known to be the principal risk factor for dengue severity). Concerning the epidemiological risk factors, a high vector density, a high virus circulation and a vulnerable population (prone to secondary infection) increase dengue severity [13]. Humidity, rainfall and the presence of water deposits and garbage favor mosquito breeding [10,14]. Finally, the serotype and the strain virulence are among viral factors [13].

Moraes and colleagues’ study [17] associates specific variables (including social variables) to an increased risk of death from dengue at an individual level. This study unveils that a higher risk of death from severe dengue is mostly present in socially disadvantaged groups (lower education level, black/brown “race/color”, rural residence), “suggesting that differences in case-fatality rates from severe dengue go beyond biological factors and enter the realm of health iniquities” [17]. Therefore, an individual’s socioeconomic position, a structural social determinant of health inequities [21], influences dengue mortality.

It is argued that living conditions, poverty, social inequalities, and illiteracy constitute the general background of dengue transmission [13]. Moreover, population density is associated with dengue mortality in Latin America and the Caribbean [18], which may indicate population growth and unplanned urbanization, both factors involved in the transmission of dengue [10]. Likewise, dengue mortality is inversely associated with the region countries’ Human Development Index, suggesting an effect of poverty, among other factors, on access to quality health care [18]. In fact, early detection and access to quality and adapted medical care can lower dengue fatality rates to less than 1% [1,2,17]. Hence, the health system acts as a social determinant of health shaping dengue mortality.

### 3. A social autopsy tool for dengue mortality

The social autopsy tool turned out to be practical and valid in the dengue mortality Colombian context after some modifications (e.g.: discarding or modifying ambiguous questions, enlarging the range of pre-formatted responses when necessary, re-wording questions when not understood). The tool may be found in S2 Appendix.

The social autopsy tool is a four-version questionnaire (fatal case/near miss; minor/adult) consisting on a 45–55 minute interview. It is designed to be administered by two trained interviewers: one to perform the interview and the other one to write down the answers and to provide additional support when needed. The tool is mostly composed by closed-ended questions in a multiple-choice format but it also includes open-ended questions (to enquire on the narration of the disease).

It aims to gather data on the following dimensions:

- Social determinants: Demographic factors (e.g. urban/rural zone), transport-related factors (e.g. access roads), environmental factors (e.g. presence of water deposits), epidemiological factors (e.g. epidemiological reporting period), living conditions and lifestyles (e.g. provision of basic services) and economic and psychosocial resources (e.g. family income).
- Individual determinants: Non-modifiable factors (e.g. age), modifiable factors (e.g. health insurance), health status (e.g. secondary dengue infection), knowledge on dengue (e.g. access to information on dengue), perceptions (e.g. self-perceived health), and individual experience with the health system (e.g. perception of health care provided).
- Health systems determinants: Service provision (e.g. population-based prevention activities), drugs and vaccines (e.g. vaccination card), funding (e.g. health care costs), governance and leadership (e.g. administrative barriers).

The tool is divided in 11 Sections. Sections 1 and 2 are related to the interview itself. Sections 3 and 4 enquire sociodemographic information on the interviewee, whether it’s the patient or the next of kin. It includes years of schooling, type of health insurance, and time of travel to nearest accessible health facility, among others. The details of the disease are narrated on a chronological order on section 5 by an open history question (“Can you tell me how the illness began?”). The interviewer prompts (with semi-open ended questions) on further details regarding behavior vis-à-vis symptoms, on treatment received, waiting times, and barriers in seeking care that the patient may have experienced. Reasons for seeking care are also inquired. Section 6 and 7 are related to the health system. Section 6 consists on filling out a table on all the health centers that the patient consulted, including means of transportation, time of travel, waiting time for treatment, treatment costs, and referral processes to other institutions. On the other hand, Section 7 looks into the interviewee’s perception of provided care (semi-open questions). All of the costs related to the illness (transportation, medical services, and personal expenses) are listed in section 8. Further on, section 9 is dedicated to list the patient’s chronic diseases, past dengue fever illness, and previous vaccination. Afterwards, section 10 enquires on patient’s access to dengue information and on the implemented population-based prevention activities. Finally, section 11 provides information on health-related behavior (e.g. having uncovered water recipients) and the living conditions (type of sanitation services, source of drinking water, methods of waste disposal, etc.).

**Preliminary results.** Although the objective of this study did not include reporting findings on the utilization of the social autopsy tool, we provide a brief example on how certain findings could be presented (table 3). Results should be interpreted with caution as they are exploratory and serve as an example only. This is a preliminary result which describes the types of delay (phase I, II, III or IV) related to dengue severity (near misses) and mortality (fatal cases) in our pilot study. This summarized table is to be interpreted along with the conceptual framework previously presented (Fig. 2). As shown in the table, neither of the near misses experienced a delay in phase IV in receiving adequate and appropriate care for dengue. On the other hand, all of the three fatal cases experienced a delay in phase IV. It is also important to note that almost all of the cases (seven out of nine) experienced a delay in phase III: in identifying and reaching the health facility that offers necessary care. This delay wasn’t however related to transportation barriers to reach health facilities. Instead, it was related to delays in receiving care (waiting times at health facility and administrative barriers related to health insurance coverage) demonstrating barriers in access to health care services. No further analysis were performed, as the sample was too small altogether (n = 9) making it impossible to compare quantitatively near misses and fatal cases. As the objective of this article is only to report the development of the tool, future work will concentrate on the utilization of the social autopsy tool on a considerable larger sample.

**Table 3:** Preliminary results.

Type of Delay				
	Phase I	Phase II	Phase III	Phase IV
NM1			Yes^1^	
NM2	Yes^1^			
NM3	Yes^1^	Yes^1^	Yes^2^	
NM4	Yes^1^	Yes^1^	Yes^2^	
NM5	Yes^1^	Yes^1^	Yes^2^	
NM6			Yes^2^	
FC1			Yes^1^ ^,^ ^2^	Yes^1^ ^,^ ^2^
FC2		Yes^1^	Yes^1^ ^,^ ^2^	Yes^1^ ^,^ ^2^
FC3				Yes^2^

^1^: Preventive care seeking

^2^: Emergency care seeking

NM: Near miss

FC: Fatal case

## Discussion

### Lessons learned on the pilot study and recommendations

The social autopsy tool is a practical method to study the social determinants of dengue mortality in the Colombian context. It produces valid results for the context that it was designed for. It is based on the scientific literature, on a validated conceptual framework and on researchers’ and health professionals’ expertise, and is now ready to be used in the main study, with some considerations to take into account.

Piloting the social autopsy tool allowed us to consider some important factors when applying the tool in a research context. The following recommendations were formulated:

- Inquire ahead of time on the applicable standards for a social autopsy study in the context in order to avoid delaying data collection.
- Consider working with partners, like with local health authorities, in order to have access to data and to give credibility to the study, as well as reassuring participants.
- Establish the importance of the study from the first (telephone) contact in order to increase the odds of the person’s participation.
- Interview merely families that have been informed of the cause of death (for fatal cases), since it is the local health authorities’ role to inform the families on the confirmed cause of death.
- Train interviewers on interview methods and provide them with support tools on addressing illness and death and handling emotionally charged moments that may emerge during the interview.
- Administrate the tool with two interviewers: one to interview and the other one to write answers in the questionnaire and to ensure that all questions are answered in the established order for a better fluidity.
- Establish a trust relationship with the family, especially for a fatal case.

### Strengths and limitations

As interpretation of a pilot study’s result should focus on practicality [38], here we describe why we consider the tool fulfills the practicality criteria previously established. It can be administrated in a reasonable time (45–55 minutes) for interviews dealing with sensitive issues; the type of questions and the answer-format are appropriate for what the tool is intended for; interviewees understand the questions asked; and it can be adapted to four cases: fatal case adult, fatal case minor, near miss adult, near miss minor. In addition, it was tested for face and content validity. While it would have been desirable to ask a small number of interviewees on their opinion on whether the tool assesses what it is supposed to, none of our interactions with interviewees underscored interpretability problems on their part. Although content validity was not assessed by an independent group of experts—which would have provided a more robust perspective- this was offset by an in-depth knowledge of the experts’ understanding of the exact context in which data is drawn from. Furthermore, the design and pre-testing of the tool were characterized by a rigorous and transparent procedure.

Although the next-of-kin of three fatal cases were not reachable, -as they had moved out or changed telephone number- 100% of the participants that were contacted via our telephone call accepted a first home-visit. This indicates that the study population, relatives of fatal cases, as well as near misses, are willing to participate on a study exploring the determinants of dengue mortality. However, the relatives of two fatal cases did not accept to pursue the interview (after the telephone call) because they were not yet notified on the confirmed cause of death. Hence, the importance to contact only confirmed cases.

Only a small number of fatal cases was included (n = 3) due to the fact that the proportion of deaths was relatively small in the metropolitan area of Bucaramanga. We tried however to compensate this fact, by adding double the number of near misses (n = 6). This is also done in maternal mortality, where the number of death cases is small, so near-misses are also considered for the study of determinants of mortality [25]. This strategic choice implied nevertheless certain limits. Interviewing a near miss does not provide the same type of information as interviewing the next of kin of a fatal case [25]. This introduces a bias as these two cases are not comparable. Interviewing a near miss generates richer information concerning the determinants of severe morbidity, as the victim of dengue fever can attest the facts of the story of the disease [39], especially concerning perceptions. This is an important bias to consider if a case-control study is to be done.

Another bias to consider is recall bias. In our study, this is an inevitable bias since the subject of interest (dengue mortality or survival) is a past event. We hence enquired retrospectively. For a future study, this bias could be measured and controlled if several cases are considered when the outcome took place three, six, and twelve months ago, in order to observe the moment where recall bias is more prominent.

### The first social autopsy tool for dengue mortality

Although a consortium of 14 partners based worldwide [46] intends to develop an effective surveillance system for epidemic dengue using novel tools, the envisioned tools are focused on laboratory diagnosis and vector monitoring (by studying the entomological and environmental indicators of dengue transmission) rather than the SDH. The tool we propose is innovative because to our knowledge, it’s the first time that a social autopsy tool has been created for the dengue mortality context. We based our work on concepts traditionally used in maternal and child health. It is also the first time that a conceptual framework on determinants of dengue mortality is to be published. The model integrates several pertinent conceptual frameworks in order to better understand the studying of the social determinants of dengue mortality.

Our social autopsy tool has the same objective as other social autopsy tools: to explore the social, behavioral, and health systems determinants of deaths [26,27]. In this sense, the tool allows the establishment of a *social diagnosis* [27] of dengue related-deaths. Our tool aims to identify the socioeconomic background of the individual, the living conditions, the care-seeking process, the experience with the health system, the barriers for an efficient access to quality care, the costs related to disease, and the knowledge on dengue transmission and consequences.

Other social autopsy tools have used the “three delays” model [47], as well as the Pathway to Survival [27] as conceptual frameworks. In our case, the social autopsy tool is based on a conceptual framework created particularly for dengue mortality and for our study. It is however inspired on an enlarged “three delays” model in order to take into account the complexity of the factors involved in health care use related to dengue mortality.

### Scope of the tool: individual vs contextual determinants

As with any reliable and valid tool, the social autopsy tool has a limited scope. The tool is not focused on all of the determinants of dengue mortality as portrayed by the conceptual framework. Indeed, the factors involved in dengue mortality are situated at different levels; some being very macroscopic, such as economic factors. Specifically, our social autopsy tool is designed to focus on the individual’s socioeconomic position and the intermediary social determinants of health. Other factors in our model should be assessed with other level-appropriate tools and methods in future studies.

One of the factors that would be worthwhile studying in the future is the socioeconomic and political context, an essential part of dengue mortality that we cannot ignore. This is because being insured in Colombia does not guarantee an efficient access to health care services [48,49], and this is fairly influenced by the socioeconomic and political context. For instance, barriers are encountered in the health system by indigenous communities as services were not planned for a culturally diverse population [48]. There is also an absence of a clear public policy in health with respect to the country’s epidemiological profile [48] leaving aside health planning based on the magnitude and the severity of dengue epidemics. Furthermore, the health system, as well as the population, is subject to market forces which creates economic barriers to provide efficient health services to the most precarious populations [48]. The consequences of these barriers results in much less attention to health needs, especially among the low-income people [49], which may result in an increase in mortality and a worsening public health [48].

More research is needed on social determinants of health, as these affect all health outcomes, including mortality. Studying the structural determinants of health inequalities on dengue mortality is a new venue, different than traditional public health approaches integrating one-dimensional views, in order to further the studying on social determinants of health and the way these are applied to neglected tropical diseases (NTDs), like dengue. Without the understanding of the root causes of NTDs, “our understanding and management of NTDs is inevitably reduced to a strategy that relies on a repetitive, reductionist, flat-world science to overcome an acknowledged complex system” [50].

### Policy implications

The information generated by the tool may provide evidences on possible future actions in both the health and the social sectors. Death reviews, like social autopsies, offer the opportunity to examine the circumstances and the contributing factors of deaths [51]. The aim of performing these reviews is to identify the modifiable death-related factors in order to design appropriate solutions to avoid future morbidity and mortality [25]. Therefore, the social autopsy tool for dengue mortality may help to better understand the structural and intermediary causes of dengue mortality and to better identify the appropriate interventions to reduce mortality.

With regard to the public health implications of a social autopsy tool for dengue mortality, it could be integrated in the Colombian surveillance system. In fact, regular meetings with health professionals take place every time there is a probable dengue-related death with the objective of confirming or excluding dengue as the cause of death and examining the delays in health care use. The analysis of death cases are mainly based on medical records; hence, integrating a tool that provides information on social variables other than clinical information, may help to better understand the contributing factors of death. For instance, interviewing individuals on their personal experience when accessing health care allows the identification of important elements that are not always reported in medical records [52], like the description of events before reaching a health care facility or the perceptions of illness.

Integrating such tool implies necessarily certain challenges. A feasibility study would have to be done in order to understand in what way a social autopsy tool could be integrated in the Colombian surveillance system. Although our experience with Santander’s Department of Health was positive, it would be necessary to explore other health professionals’ receptiveness towards a tool like this, since biomedical scientists generally exhibit a negative posture towards the social sciences [53]. This posture is mainly founded on the methodological approach used in social sciences (non-experimental designs) [53]. We have however demonstrated how a social autopsy tool may be rigorously developed and validated. Such tool is pertinent in order to provide complementary information and allow a global analysis of dengue mortality. In fact, death reviews in social sciences may contribute to broader debates about healthcare and considerate the social, economic and political dynamics for delivery of care [52]. All of this, with the purpose of better identifying the modifiable and avoidable factors in order to better formulate the interventions to reduce dengue mortality.

## Supporting Information

S1 Appendix Indicators of the determinants of dengue mortality identified by the social autopsy tool. (DOCX)

S2 Appendix Social autopsy tool (fatal case minor, Spanish version CF18-). (DOCX)
