# Supplementary material for: Developing a Social Autopsy Tool for Dengue Mortality: A Pilot Study
Source: PLoS One. 2015 Feb 6;10(2):e0117455. doi: 10.1371/journal.pone.0117455 (PMC4320105; doi:10.1371/journal.pone.0117455)
Supplement: S1 Appendix — (DOCX) [file pone.0117455.s001.docx]

**Appendix S1. Indicators of the determinants of dengue mortality identified by the social autopsy tool**

| DIMENSION | | INDICATOR | TOOL | | | |
| --- | --- | --- | --- | --- | --- | --- |
|  | | | Interview  (SA tool) | Observation (SA tool) | Medical Records | Other |
| SOCIAL DETERMINANTS | | | | | | |
| 1. Demographic Factors (DEM) | Urban/rural zone | | X  *(Section 4)* |  |  |  |
|  | Place of residence | | X *(Section 4)* |  |  |  |
|  | Informal settlement | |  | X  *(Section 4)* |  | X |
|  | Migration | | X  *(Section 4)* |  |  | X |
|  | Population growth | |  |  |  | X |
|  | Average time (distance) to closest available health facility | | X *(Section 4)* |  |  |  |
| 2. Transportation (TRAN) | Means of transportation | | X *(Section 4 & 6)* |  |  |  |
|  | Transportation costs (related to illness) | | X  *(Section 8)* |  |  |  |
|  | Access routes | |  | X  *(Section 11)* |  |  |
| 3. Environmental Factors (AMB) | Temperature, humidity, rainfall | |  |  |  | X |
|  | Water retention sources (lakes, rivers, etc.) | | X  *(Section 11)* |  |  |  |
| 4. Epidemiological Factors (EPI) | Age of endemicity | |  |  |  | X |
|  | Epidemiological period of notification | |  |  |  | X |
|  | Travel to endemic area | | X  *(Section 4)* |  |  |  |
| 5. Living Conditions and Lifestyle (COND) | Provision of basic services (water, waste disposal) | | X  *(Section 11)* |  |  |  |
|  | Number of rooms in household | | X  *(Section 11)* |  |  |  |
|  | Main material composition (floor, roof, walls) | | X  *(Section 11)* |  |  |  |
|  | Peridomiciliary sanitation | |  | X  *(Section 11)* |  |  |
|  | Water deposits (Water tanks outside the house and at ground level) | | X  *(Section 11)* | X  *(Section 11)* |  |  |
|  | Methods to control mosquitoes | | X  *(Section 11)* | X  *(Section 11)* |  |  |
| 6. Political Factors (POL) | Congruence of social actions with cultural reality | |  |  |  | X |
|  | Political commitment for the development of prevention and dengue control policies and programmes | |  |  |  | X |
|  | Intersectoral collaboration | |  |  |  | X |
| 7. Individual Resources (economic, psychosocial, etc.) (REC) | Therapeutic savoir-faire (traditional practices, auto medication, etc.) (intake of fluids and fever control) | | X *(Section 5)* |  |  |  |
|  | Available resources in the biopsychosocial environment | | X *(Section 5)* |  |  |  |
|  | Family income | | X  *(Section 11)* |  |  |  |
|  | Attitudes and environmental influences (for family decision making)  - Attitudes towards dengue  - Social support networks | | X *(Section 5)* |  |  |  |
| INDIVIDUAL DETERMINANTS | | | | | | |
| 1. Non-modifiable Factors (NOM) | | Age | X *(Section 3)* |  | X |  |
|  |  | Sex | X  *(Section 3 & 4)* |  |  |  |
|  |  | Ethnic group | X  *(Section 3 & 4)* |  |  |  |
| 2. Modifiable Factors  (MODI) | | Marital status | X  *(Section 3 & 4)* |  |  |  |
|  |  | Population group | X  *(Section 3 & 4)* |  |  |  |
|  |  | Religion | X  *(Section 3 & 4)* |  |  |  |
|  |  | Literacy | X  *(Section 3 & 4)* |  |  |  |
|  |  | Formal education | X  *(Section 3 & 4)* |  |  |  |
|  |  | Family composition (ex: head of household) | X  *(Section 11)* |  |  |  |
|  |  | Autonomy | X  *(Section 4)* |  |  |  |
|  |  | Occupation | X  *(Section 3 & 4)* |  |  |  |
|  |  | Spouse’s occupation | X  *(Section 4)* |  |  |  |
|  |  | Individual income | X  *(Section 3 & 4)* |  |  |  |
|  |  | Capacity to pay (related to illness) | X  *(Section 8)* |  |  |  |
|  |  | Social (health) insurance (contributive, subsidized, …) | X  *(Section 3 & 4)* |  |  |  |
| 3. Health Conditions (SAL) | | Pregnancy at time of disease | X  *(Section 4)* |  | X |  |
|  |  | Pre-existing conditions / Chronic health problems (diabetes, hypertension, kidney disease, asthma, leukemia, cancer, HIV…) (smoking, drugs, alcohol) | X  *(Section 9)* |  | X |  |
|  |  | Secondary infection with different serotype / History of dengue infection |  |  |  | X (Labo) |
|  |  | Interval between first and second infection |  |  |  | X (labo) |
|  |  | Sequentiality of serotypes in secondary infections |  |  |  | X (Labo) |
| 4. Knowledge (CON) | | Access to knowledge on dengue (community, media, etc.) | X  *(Section 10)* |  |  |  |
|  |  | Knowledge on consequences of dengue | X  *(Section 10)* |  |  |  |
| 5. Perceptions (PERC) | | Self-reported health | X  *(Section 5)* |  |  |  |
|  |  | Subjective assessment of the disease / Risk perception | X  *(Section 5)* |  |  |  |
| 6. Individual’s Experience with the Health System  (ISS) | | History of access to health services  - Previous health services consultation  - Previous health services consultation for dengue  - History of hospitalization | X *(Section 4 & 9)* |  | X |  |
|  |  | The patient’s / family’s perception of care before and after complications (preventive care, emergency care)  - Satisfaction of care received  - Perception of patient-doctor relationship | X  *(Section 7)* |  |  |  |
| HEALTH SYSTEMS DETERMINANTS | | | | | | |
| 1. Service provision (PRES) | | Level of care of health facility |  |  | X |  |
|  |  | Quality of preventive care –  Identification and management of early signs of severity  - Diagnostic laboratory tests for dengue  - History of previous dengue infection  - Number of early symptoms of severity (headache, exanthema, nausea/vomit, myalgia, arthralgia, diarrhea…)  - Tourniquet test results  - Mild bleeding  - Time interval between onset of symptoms and case notification  - Case management in groups A, B, or C |  |  | X | X |
|  |  | Quality of emergency care – Identification and management of late signs of severity  - Hematuria  - Upper gastrointestinal bleeding  - Number of warning signs (severe abdominal pain, hypovolemic shock, hepatic failure…)  - High haematocrit,  - Low platelet count,  - Cavitary effusion  - Case management in groups A, B, or C |  |  | X |  |
|  |  | Public health population-based interventions (health education, awareness campaigns, fumigation…) | X *(Section 10)* |  |  | X |
| 2. Health Personnel  (PERS) | | Adequate health personnel available (doctors, nurses, etc.) |  |  | X |  |
|  |  | Training of health workers |  |  |  | X |
| 3. Health Information (INFO) | | Quality of the patient’s medical record |  |  | X |  |
|  |  | Quality of dengue case registration |  |  |  | X |
|  |  | Surveillance system |  |  |  | X |
| 4. Drugs and vaccines (MED) | | Drug availability |  |  |  | X |
|  |  | Drug costs |  |  |  | X |
|  |  | History of vaccination and costs (access to vaccines by the “Entidades Promotoras de Salud”) | X  *(Section 9)* |  |  | X |
| 5. Funding (FIN) | | Private – public funding |  |  |  | X |
|  |  | Social security (health insurance) |  |  |  | X |
|  |  | User fees for health care | X  *(Section 6 & 8)* |  |  | X |
| 6. Governance and leadership  (GOB) | | Administrative barriers (by EPSs) (opening hours, waiting times for medical appointments, reasons approved for consultation…) | X *(Section 5 & 6)* |  |  | X |
|  |  | Persistence of biological reductionist models |  |  |  | X |
|  |  | Curative-focused system vs. preventive-focused system |  |  |  | X |
