# Supplementary material for: Developing a Social Autopsy Tool for Dengue Mortality: A Pilot Study
Source: PLoS One. 2015 Feb 6;10(2):e0117455. doi: 10.1371/journal.pone.0117455 (PMC4320105; doi:10.1371/journal.pone.0117455)
Supplement: S2 Appendix — (DOCX) [file pone.0117455.s002.docx]

**Appendix S2. Social autopsy tool (fatal case minor, Spanish version CF18-)**

Código entrevista: AS____

**CENTRO DE ATENCION Y DIAGNOSTICO DE ENFERMEDADES INFECCIOSAS E INFOVIDAS**

| **Sección 1: Datos de la entrevista** | | |
| --- | --- | --- |
|  | Nombre del/de la entrevistador/a | Nombres: Apellidos: Acompaña: |
|  | Fecha del primer intento de entrevista | Día ☐ Mes ☐ Año ☐  Código: __________ |
|  | Fecha del segundo intento de entrevista | Día ☐ Mes ☐ Año ☐  Código: __________ |
|  | Fecha de la entrevista | Día ☐ Mes ☐ Año ☐  Código: __________ |
|  | Hora de inicio de la entrevista | Hora: ____ Minuto: ____ |
|  | Hora final de la entrevista | Hora: ____ Minuto: ____ |
|  | Códigos para entrevista  1. Completada 2. Parcialmente completada 3. Entrevistado pospuso entrevista 4. Ningún entrevistado elegible en el momento de la visita 5. Entrevistado elegible rechazó entrevista | 6. Ningún entrevistado elegible vive en la vivienda 7. Ningún miembro del hogar está en la vivienda (vivienda cerrada) 8. Vivienda deshabitada o no encontrada 9. No existe persona que dé información (paciente consultó solo) 🡪 Insistir hasta encontrar información |
|  | *[Entrevistador, llene la descripción de la categoría a la cual el paciente pertenece.]* | ☐ Caso fatal adulto ☐ Caso fatal menor de edad o dependiente ☐ Control (near miss) adulto ☐ Control (near miss) menor de edad o dependiente |

| **Sección 2: Inicio de la entrevista y consentimiento** | | |
| --- | --- | --- |
| *Instrucciones: Presentarse y explicar el objetivo de la visita. Preguntar si se puede hablar con la persona más cercana que estuvo durante la enfermedad del paciente.  Ejemplo:  “Mi nombre es _______. Soy la enfermera del estudio “Autopsia social – Complicaciones y mortalidad por dengue”. Me han informado que hubo un caso de dengue en esta familia, lo cual lo sentimos mucho. Por lo tanto, le haremos una entrevista con el fin de conocer algunos detalles sobre la enfermedad del dengue para evaluar y poder mejorar los servicios de salud y los programas de salud pública.”* | | |
| **2.1** | Consentimiento  *[Leer el formulario de consentimiento. Preguntar si entrevistado tiene preguntas. Preguntar si desea continuar con la entrevista.]* | Si ☐  No ☐ |

| Sección 3: Datos del entrevistado | | | |
| --- | --- | --- | --- |
|  | Nombres y apellidos del entrevistado | | Iniciales: |
|  | Sexo del entrevistado | | Femenino ☐ Masculino ☐ |
|  | Fecha de nacimiento del entrevistado | | Día ☐ Mes ☐ Año ☐ |
|  | “Cuál era su relación con el/la niño/a?” | | ☐Esposo/a ☐Hijo/hija  ☐Hermano/a ☐Madre/padre ☐Abuelo/abuelo ☐Suegro/suegra ☐Nieto/a ☐Tío/tía ☐Nuera/yerno ☐Amigo/a  ☐Vecino/a ☐Otro________________ |
| 1. **a**   **3.5b** | *[Entrevistador: Notar si otras personas presentes en la entrevista.]*  “De las personas aquí presentes, quién acompañó al/ a la niño/a durante la enfermedad?” | | ☐ Sí (ver pregunta 3.5b) ☐ No, el entrevistado es el único presente   \|  \| Presente en la entrevista \| Acompañó al paciente en momento de enfermedad \| \| --- \| --- \| --- \| \| a) Esposo/a \| ☐ \| ☐ \| \| b) Hijo/hija \| ☐ \| ☐ \| \| c) Hermano/a \| ☐ \| ☐ \| \| d) Madre/padre \| ☐ \| ☐ \| \| e) Abuela/abuelo \| ☐ \| ☐ \| \| f) Suegro/suegra \| ☐ \| ☐ \| \| g) Nieto/a \| ☐ \| ☐ \| \| h) Tío/tía \| ☐ \| ☐ \| \| i) Nuera/yerno \| ☐ \| ☐ \| \| j) Amigo/a \| ☐ \| ☐ \| \| k) Vecino/a \| ☐ \| ☐ \| \| l) Otro__________ \| ☐ \| ☐ \| |
|  | MODI | “Cuál es su estado civil?” | ☐ Soltero/a ☐ Casado/a ☐ Unión libre ☐ Viudo/a ☐ Divorciado/a ☐ Desconoce |
|  | NOM | “A qué etnia pertenece usted?” | ☐ Mestiza ☐ Blanca ☐ Negra ☐ Asiática  ☐ Indígena ☐ Otra |
|  | MODI | “Me puede indicar si usted era discapacitado, migrante, gestante, desplazado o carcelario al momento de la enfermedad del/de la niño/a?” | ☐ Discapacitados ☐ Desplazados ☐ Migrantes ☐ Carcelarios ☐ Gestantes ☐ Ninguno (el resto)  ☐ Desconoce |
|  | MODI | “Qué religión profesa la familia?” | ☐ Católico ☐ Evangélico ☐ Protestante ☐ Mormón ☐ Testigo de Jehová ☐ Adventista ☐ Judío ☐ Musulmán  ☐ Ateo/agnóstico ☐ Ninguna ☐ Otra____________ ☐ Desconoce |
|  | MODI | “Usted sabe leer y escribir?” (al momento de la enfermedad del niño/a) | ☐ Sí  ☐ No ☐ Desconoce |
|  | MODI | “Hasta que año de escolaridad cursó usted?” (al momento de la enfermedad del niño/a) | ☐ No asistió  ☐ Primaria, año: ___________ ☐ Secundaria, año: ___________ ☐ Universidad, semestre: __________  ☐ Desconoce |
|  | MODI | “Cuál era su ocupación en el momento en el que el/la niño/a se enfermó?” | ☐ Empleado ☐ Desempleado ☐ Pensionado/a ☐ Estudiante ☐ Estudiante y empleado ☐ Independiente |
|  | MODI | “Durante el último año, antes de la enfermedad del/de la niño/a, cuál era su ingreso mensual?”  *[El salario mínimo es 589.500 pesos]* | ☐ Dependiente  ☐ Menos que el salario mínimo mensual vigente ☐ Igual al salario mínimo mensual vigente  ☐ Entre 1 y 2 salarios mínimos mensuales  ☐ Entre 3 y 4 salarios mínimos mensuales  ☐ Más de 5 salarios mínimos mensuales  ☐ Desconoce |
|  | MODI | “Cuál es su tipo de régimen de seguridad social en salud?” | ☐ Contributivo ☐ Subsidiado ☐ Excepción ☐ Especial ☐ No afiliado ☐ Desconoce |

| Sección 4 : Datos de identificación del paciente | | | |
| --- | --- | --- | --- |
|  | DEM | Dirección de la vivienda | Dirección: Barrio: Municipio: Departamento: |
|  | DEM | Zona *[Entrevistador: observar y notar]* | ☐ Urbana  ☐ Rural  ☐ Vereda |
|  | DEM | Asentamiento informal  *[Entrevistador: observar y notar]* | ☐ Sí Nombre: ☐ No Número de casa: |
|  |  | Identificación niño/a | Iniciales: |
|  | NOM | Sexo del/de la niño/a | Femenino ☐ Masculino ☐ |
|  | NOM | “A qué etnia pertenecía el/la niño/a?” | ☐ Mestiza ☐ Blanca ☐ Negra ☐ Asiática  ☐ Indígena ☐ Otra |
|  | MODI | “Me puede indicar si el/la niño/a era discapacitado, migrante, gestante, desplazado o carcelario al momento de enfermarse?” | ☐ Discapacitados ☐ Desplazados ☐ Migrantes ☐ Carcelarios ☐ Gestantes ☐ Ninguno (el resto)  ☐ Desconoce |
|  | MODI | “El/la niño/a sabía leer y escribir al momento de enfermarse?” | ☐ Sí  ☐ No ☐ Desconoce |
|  | MODI | “Hasta que año de escolaridad cursó el/la niño/a?” (al momento de enfermarse) | ☐ No asistió  ☐ Primaria, año: ___________ ☐ Secundaria, año: ___________ ☐ Universidad, semestre: __________  ☐ Desconoce |
|  | MODI | “El/la niño/a estaba matriculado como estudiante al momento de enfermarse?” | ☐ Sí  ☐ No ☐ Desconoce |
|  | MODI | “Cuál era el tipo de régimen de seguridad social en salud del/de la niño/a al momento de enfermarse?” | ☐ Contributivo ☐ Subsidiado ☐ Excepción ☐ Especial ☐ No afiliado ☐ Desconoce |
|  |  | Nombre EPS | _______________________ |
|  |  | Nombre ARS | _______________________ |
|  | DEM | “A cuánto tiempo de su vivienda se encuentra la IPS o el centro de atención de salud más cercano que podía atender al/ a la niño/a?”  “Por qué medio de transporte?” | ☐ 0-15 min ☐ 16-30 min ☐ 31-45 min ☐ 46-60 min ☐ 1h01-1h30 hora ☐ 1h31-2h ☐ 2h01 o más ☐ Desconoce  ☐ A pie ☐ Bicicleta ☐ Bus ☐ Taxi ☐ Moto ☐ Auto compartido  ☐ Auto propio ☐ Otro ____________ |
|  | ISS | “En caso de enfermedad aguda, a qué institución de salud acudía el/la niño/a normalmente?” (ej.: enfermedad diarreica aguda, infección respiratoria aguda)  Cuánto tiempo se demoraba en llegar allá normalmente?  *[Preguntar sólo si se aplica]*  “Si la institución de salud que acudía cuando el/la niño/a se enfermaba era más lejana, por qué no asistía a la más cercana?” | ☐ Hospital ☐ Clínica ☐ Consultorio privado ☐ Puesto de salud  ☐ Droguería/consultorio ☐ Otro ___________ ☐ Desconoce ☐ Ninguno  ☐ 0-15 min ☐ 16-30 min ☐ 31-45 min ☐ 46-60 min ☐ 1h01-1h30 hora ☐ 1h31-2h ☐ 2h01 o más ☐ Desconoce  _____________________________________  _________________________________________________________________________________________________________________________________________________________________________________________ |
|  | MODI aut | “Generalmente quién tomaba la decisión de consultar los servicios de salud?” | ☐ El paciente mismo ☐ El/La esposo/a del paciente ☐ Los padres del paciente ☐ Otros familiares del paciente ☐ Otro ___________________  ☐ Desconoce |
|  | DEM | “La familia inmigró a esta ciudad (antes de la enfermedad del/ de la niño/a)?”  *[Si respuesta afirmativa, pregunte dónde vivía antes, para asegurarse que fue una migración]* | ☐ Sí, migró ☐ No migró ☐ Desconoce |
|  | EPI | “Sabe usted si el niño/a estuvo en otra ciudad los últimos 15 días antes de enfermarse?”  *[Si respuesta afirmativa, liste las ciudades]* | ☐ Sí ________________________________  ☐ No estuvo ☐ No recuerda/Desconoce |

| Sección 5: Características de la enfermedad, proceso de búsqueda de atención, de tratamientos proporcionados y de motivos |
| --- |
| *[Es importante que en esta parte el entrevistado no se sienta juzgado, ya que se puede sentir sensible a la crítica. Es importante demostrar que uno está interesado en las barreras para consultar y no en juzgar al entrevistado.]* |
| 1. **“Quién acompañó al/a la niño/a durante la enfermedad?”** ___________________________________________ 2. **“Recuerda qué fecha inició la enfermedad?”**   Fecha: _________________________________   1. **“Cuántos días estuvo enfermo/a el/la niño/a?** _________________________________ 2. **“A los cuántos días consultó después de la aparición de los primeros síntomas?”**   _____________________________ |
| [Apoyarse en la siguiente lista de síntomas para ayudar a recordar a la persona los síntomas que el niño/a tuvo]   - Fiebre - Dolor de cabeza - Dolor en los ojos - Diarrea - Náuseas - Escalofríos - Malestar general - Vómitos - Dolores musculares - Dolores articulares - Puntitos rojos en todo el cuerpo (Petequias) - Tenía los ojos amarillos (ictericia) - Le dijeron si tenía los glóbulos blancos bajos - Supo usted si tenía las plaquetas bajas - Dolor abdominal intenso - Vómito persistente - Sangrado de mucosas - Letargia y/o irritabilidad - Dificultad respiratoria - Alteración de la conciencia |
| 1. **“Me puede contar cómo empezó la enfermedad?”**   *[Preguntar en orden cronológico mañana, tarde, noche, primer día de la enfermedad, segundo día, etc. Apoyarse en frases como: “Y después, qué pasó?” “Que más pasó?” “Qué más recuerda?”]*  ***Preguntar por:***   - Síntomas - Qué hizo y por qué - Tratamientos proporcionados dentro y fuera de la casa - Quién trató al niño o a la niña, dónde - Por qué acudió o por qué no acudió servicios de salud - Cuántos días pasaron hasta consultar servicios de salud - Tratamientos proporcionados en servicios de salud, qué le dijeron - Remitieron o no, a dónde y por qué - Trámites con el seguro, pagos o co-pagos para ser tratado - Problemas administrativos en la IPS y con la EPS |

________________________________________________________________________________________________________________________________________________________________________________________________________________________________________________________________________________________________________________________________________________________________________________________________________________________________________________________________________________________________________________________________________________________________________________________________________________________________________________________________________________________________________________________________________________________________________________________________________________________________________________________________________________________________________________________________________________________________________________________________________________________________________________________________________________________________________________________________________________________________________________________________________________________________________________________________________________________________________________________________________________________________________________________________________________________________________________________________________________________________________________________________________________________________________________________________________________________________________________________________________________________________________________________________________________________________________________________________________________________________________________________________________________________________________________________________________________________________________________________________________________________________________________________________________________________________________________________________________________________________________________________________________________________________________________________________________________________________________________________________________________________________________________________________________________________________________________________________________________________________________________________________________________________________________________________________________________________________________________________________________________________________________________________________________________________________________________________________________________________________________________

| **Sección 6 : Consulta de servicios de salud (Ver tabla 2)** |
| --- |
| *[Escribir en la columna INSTITUCIÓN DE SALUD la lista de los prestadores de servicios que el paciente consultó en orden (hospital, laboratorio, clínica, etc.) No incluye droguería. Repetir las siguientes preguntas por cada institución de salud y llenar tabla]* |

| **TABLA 2** | | **INSTITUCIÓN DE SALUD** | | | | |
| --- | --- | --- | --- | --- | --- | --- |
| **PREGUNTAS** | | Institución 1   _______________ | Institución 2  ________________ | Institución 3  _____________ | Institución 4  ______________ | Institución 5  ____________ |
|  | “Cómo se transportó el/la paciente para ir a la institución de salud?” |  |  |  |  |  |
|  | “Cuánto tiempo se demoró en llegar a la institución de salud?”  *[En caso de haber sido referido por otra institución anteriormente, preguntar]*  “Cuánto tiempo se demoró en llegar a la institución de salud desde el momento en que lo refirieron?” |  |  |  |  |  |
|  | “Cuánto tiempo después de haber llegado a la institución de salud, obtuvo la atención o el tratamiento el/la paciente? |  |  |  |  |  |
|  | Si es que el/la paciente no fue atendido/a, o tratado/a: “Por qué la institución de salud no lo/la atendió?” |  |  |  |  |  |
|  | “Tuvo que pagar para la atención/tratamiento recibida/o?” | ☐ SI  ☐ NO  ☐ NS | ☐ SI  ☐ NO  ☐ NS | ☐ SI  ☐ NO  ☐ NS | ☐ SI  ☐ NO  ☐ NS | ☐ SI  ☐ NO  ☐ NS |
|  | “En esta institución de salud, el/la paciente fue referido/a a otra institución de salud?” | ☐ SI  ☐ NO  ☐ NS | ☐ SI  ☐ NO  ☐ NS | ☐ SI  ☐ NO  ☐ NS | ☐ SI  ☐ NO  ☐ NS | ☐ SI  ☐ NO  ☐ NS |
|  | “A dónde fue referido el/la paciente?” |  |  |  |  |  |
|  | “Cuál fue el motivo para referir al/a la paciente?” |  |  |  |  |  |
|  | “El/la paciente acudió a la institución de referencia?”  Si la respuesta es NO, “por qué el/la paciente no acudió?” | ☐ SI  ☐ NO  ☐ NS | ☐ SI  ☐ NO  ☐ NS | ☐ SI  ☐ NO  ☐ NS | ☐ SI  ☐ NO  ☐ NS | ☐ SI  ☐ NO  ☐ NS |

| **Sección 7: Experiencia con el sistema de salud** | | | |
| --- | --- | --- | --- |
|  | ISS | “Cómo piensa usted que fue la atención de los servicios de salud?”  _______________________________________________________________ __________________________________________________________ __________________________________________________________ __________________________________________________________ __________________________________________________________ __________________________________________________________ __________________________________________________________ __________________________________________________________  _______________________________________________________________ | |
|  | ISS | “Quién fue la primera persona que atendió al/ a la niño/a en los servicios de salud?” | ☐ Enfermera ☐ Médico  ☐ Especialista ☐ Estudiante de medicina ☐ Estudiante de enfermería ☐ Otro______________ |
|  | ISS | “Qué actitud por parte del personal de salud percibió cuando el/la niño/a fue atendido/a?”  __________________________________________________________ __________________________________________________________ __________________________________________________________ __________________________________________________________ __________________________________________________________  __________________________________________________________ | |

| **Sección 8: Costos** | | | | | | | | |  |
| --- | --- | --- | --- | --- | --- | --- | --- | --- | --- |
|  | | TRAN | | | “Cuánto tuvo que pagar usted y su familia para gastos de transporte relacionados con la enfermedad del/de la niño/a?” | | | ________________$ |  |
|  | | FIN | | | “Cuánto tuvo que pagar usted y su familia por los servicios de salud por la enfermedad del/de la niño/a?” (Laboratorio, hospitalización, consulta, etc.) | | | ________________$ |  |
|  | | REC | | | “Cuánto pagó usted y su familia por otros gastos relacionados con la enfermedad del/de la niño/a?” (Hotel, alimentación, aseo, etc.) | | | ________________$ |  |
|  | | FIN REC | | | “Tuvo que endeudarse usted o su familia con terceros o dejar de pagar algún servicio de la vivienda para poder costear el tratamiento y/o gastos ocasionados por la enfermedad del/de la niño/a?” | | | ☐ Sí  ☐ No ☐ Desconoce |  |
| **Sección 9: Antecedentes del paciente** | | | | | | | | |  |
|  | SAL | | “El/La niño/a presentaba alguna de las siguientes condiciones?” | | | Si No NS  ☐ ☐ ☐ Hipertensión arterial  ☐ ☐ ☐ Diabetes  ☐ ☐ ☐ Enfermedades cardiacas  ☐ ☐ ☐ Enfermedad cerebrovascular  ☐ ☐ ☐ Enfermedad renal  ☐ ☐ ☐ Asma/EPOC  ☐ ☐ ☐ Tuberculosis  ☐ ☐ ☐ Cáncer  ☐ ☐ ☐ VIH/SIDA  ☐ ☐ ☐ Tabaquismo  ☐ ☐ ☐ Alcoholismo  ☐ ☐ ☐ Drogadicción  ☐ ☐ ☐ Obesidad  ☐ ☐ ☐ ¿ Otra enfermedad crónica? _______________________ | | |  |
| **a.**  **b.** | SAL  ISS | | “Sabe usted si el/la niño/a alguna vez se enfermó de dengue antes de esta última vez?”   Si la respuesta es afirmativa, “sabe de qué tipo de dengue se enfermó?”  Si la respuesta es afirmativa, “el/la niño/a consultó a los servicios de salud?” | | | ☐ Sí  ☐ No ☐ Desconoce Cuándo __________________________  ☐ Dengue  ☐ Dengue hemorrágico/Dengue grave  ☐ Sí  ☐ No ☐ Desconoce Cuándo __________________________ | | |  |
| **a.** | ISS | | “El/La niño/a fue alguna vez hospitalizado/a antes de que se enfermara por dengue?”  Si la respuesta es afirmativa, “cuál fue el motivo de hospitalización?” | | | ☐ Sí  ☐ No ☐ Desconoce Cuándo __________________________  Motivo: ___________________________ | | |  |
|  | MED | | “El/La niño/a recibió el esquema de vacunación?”  “Sabe si tuvo que pagar por las vacunas?” | | | ☐ Sí  ☐ No ☐ Desconoce  ☐ Sí  ☐ No ☐ Desconoce | | |  |
|  | MED SAL | | “Sabe si el/la niño/a fue vacunado/a por fiebre amarilla?” | | | ☐ Sí  ☐ No ☐ Desconoce | | |  |
| **Sección 10: Conocimientos** | | | | | | | | | |
|  | | CON | | “Ha tenido acceso a información sobre el dengue?”  Si respuesta afirmativa, “de qué manera?” | | | ☐ Sí ☐ No ☐ Desconoce  ☐ Radio ☐ Televisión ☐ Folleto ☐ Colegio ☐ Personal de salud en comunidad ☐ Personal de salud en IPS ☐ Otro _________________ | | |
|  | | CON | | “Ha tenido información sobre las complicaciones del dengue?” | | | ☐ Sí ☐ No ☐ Desconoce | | |
|  | | CON | | “Antes de la enfermedad del niño/a, usted sabía sobre las complicaciones del dengue?” | | | ☐ Sí  ☐ No ☐ Desconoce | | |
|  | | CON | | “Antes de la enfermedad del niño/a, usted sabía que se puede morir por dengue?” | | | ☐ Sí  ☐ No ☐ Desconoce | | |
|  | | PRES | | “Antes de que el/la niño/a se enfermara por dengue, hubo presencia por parte del gobierno en alguna actividad para prevenir el dengue?”   Si respuesta afirmativa, “qué tipo de actividad?” | | | ☐ Sí  ☐ No ☐ Desconoce  ☐ Fumigación ☐ Campañas masivas de salud (radio, televisión, etc.) ☐ Folleto ☐ Otro ___________________ | | |

| Sección 11: Datos de la vivienda y condiciones de vida (lugar donde el/la niño/a vivía) | | | | | |
| --- | --- | --- | --- | --- | --- |
|  | MODI | | “Cuántas personas residen en esta vivienda?”  “Qué edad tienen?” | | ☐ Total de personas  ☐ Menor de 5 años ☐ Entre 5 y 18 años ☐ Entre 19 y 64 años ☐ Mayor de 65 años |
|  | MODI | | “Quién es la cabeza de la familia?” (jefe del hogar)  Sexo | | _________________  Femenino ☐ Masculino ☐ |
|  | REC | | “Los salarios de este hogar son:  *[El salario mínimo es 589.500 pesos]* | | ☐ Nulo  ☐ Menos del salario mínimo mensual vigente ☐ El salario mínimo mensual vigente  ☐ Entre 1 y 2 salarios mínimos mensuales vigente  ☐ Entre 3 y 4 salarios mínimos mensuales vigente  ☐ Más de 5 salarios mínimos mensuales vigente”  ☐ Desconoce |
|  | COND | | “Cuántos dormitorios tiene esta vivienda?” (utilizados para dormir) | | ☐ Dormitorios |
|  | COND | | “Qué tipo de servicios sanitarios posee esta vivienda?” | | ☐ Inodoro dentro de la vivienda  ☐ Inodoro fuera de la vivienda  ☐ Batería sanitaria dentro de la vivienda ☐ Batería sanitaria fuera de la vivienda ☐ Letrina  ☐ Hoyo ☐ No hay inodoro (río, arbusto, etc.) /campo abierto ☐ Otro _________________ |
|  | COND | | “Esta vivienda tiene agua potable?” | | ☐ Sí ☐ No |
|  | COND | | “Cuál es la principal fuente de agua para el consumo humano?” | | ☐ Agua de la llave ☐ Agua embotellada ☐ Agua suministrada por camión ☐ Cisterna de agua (subterráneo)  ☐ Agua de superficie (río, lago, etc.) ☐ Agua de la lluvia ☐ Otro __________________ |
|  | AMB | | “Hay fuentes importantes de agua cerca de esta vivienda?” Ejemplo: lagos, ríos, piscinas. | | ☐ Sí ☐ No |
|  | COND | | “Cuál es el método principal de eliminación de residuos (basura)?” | | ☐ Botadero de basura/basurero ☐ Río  ☐ Calle ☐ Zanja/Fosa/Alcantarillado ☐ Hoyo privado ☐ Hoyo público ☐ Servicios de eliminación de desechos/recolección de basura ☐ Combustión/quemar  ☐ Ningún lugar designado/Por todas partes ☐ Otro _________________________ |
| ***[Para las siguientes preguntas, pedir permiso al entrevistado para observar algunas características de la vivienda]*** | | | | | |
|  | | COND | | *[Entrevistador: Observar la presencia de residuos alrededor de la vivienda (peridomicilio)]* | ☐ Sí hay residuos  ☐ No hay residuos |
|  | | COND | | *[Entrevistador: Observar de qué material está hecho el piso.]* | ☐ Tierra (lodo, arena) ☐ Cemento ☐ Tablas de madera ☐ Madera pulida/lustrada ☐ Baldosa/cerámica  ☐ Otro __________________ |
|  | | COND | | *[Entrevistador: Observar de qué material está hecho el techo.]* | ☐ Láminas de zinc (metal) ☐ Tejas ☐ Láminas de eternit ☐ Plancha (cemento)  ☐ Paja ☐ Carpas (plástico) ☐ Cartón ☐ Madera |
|  | | COND | | *[Entrevistador: Observar de qué material están hechas las paredes.]* | ☐ Láminas de zinc (metal)  ☐ Ladrillo ☐ Madera  ☐ Lodo/tierra ☐ Cartón  ☐ Paja ☐ Cemento |
|  | | TRAN | | *[Entrevistador: Observar las vías de acceso hacia la vivienda.]* | ☐ Carretera/Autopista ☐ Calle pavimentada  ☐ Calle no pavimentada ☐ Camino de tierra  ☐ No hay calle ☐ Otro__________  ☐ Vías con riesgo de inundación |
|  | | COND | | “Me podría decir si es que en la vivienda generalmente hay:   *[verificar con observación]*  *En el caso afirmativo*, “Éstos están generalmente tapados o no?” | \|  \| SÍ \| NO \| \| --- \| --- \| --- \| \| Tanques o pilas de agua fuera de la vivienda  Tanques o pilas de agua dentro de la vivienda  Floreros  Otros recipientes con agua” \| ☐  ☐  ☐ ☐ \| ☐  ☐ ☐ ☐ \|   ☐ Sí están tapados ☐ No están tapados |
|  | | COND | | “En la vivienda generalmente se usa:?”  *[Verificar con observación]* | \|  \| SÍ \| NO \| \| --- \| --- \| --- \| \| Toldillos/mosquiteros Angeos  Mallas en las puertas Otro ___________________ \| ☐☐☐ ☐ \| ☐ ☐ ☐ ☐ \| |
|  | | COND | | “Qué métodos caseros utilizan para controlar los mosquitos?” | ☐ Insecticidas ☐ Raqueta/malla eléctrica  ☐ Otro ___________________ ☐ Ninguno |
